# Brazilian front-of-package nutrition labelling and food additives: an approach to identify ultra-processed food products

**DOI:** 10.1017/S000711452500090X

**Published:** 2025-06-14

**Authors:** Daniela Silva Canella, Ana Paula Bortoletto Martins, Mariana Ribeiro, Giovanna Calixto Andrade, Vanessa dos Santos Pereira Montera, Laís Amaral Mais

**Affiliations:** 1 Institute of Nutrition, Rio de Janeiro State University (UERJ), Rio de Janeiro, Brazil; 2 Center for Epidemiological Research in Nutrition and Health (Nupens), University of São Paulo (USP), São Paulo, Brazil; 3 Institute for Consumers Defense (Idec), São Paulo, Brazil; 4 Postgraduate Program in Food, Nutrition and Health, Rio de Janeiro State University (UERJ), Rio de Janeiro, Brazil

**Keywords:** Additives, Front-of-package labelling, Ultra-processed food products, Non-sugar sweeteners, Flavourings, Colourings

## Abstract

This study aimed to explore combinations of the Brazilian front-of-package nutrition labelling (FoPNL) (high in added sugar, saturated fat or sodium) and/or three specific food additives with cosmetic functions (colourings, flavourings and non-sugar sweeteners) in packaged foods and beverages marketed in Brazil. This approach intends to strengthen the identification of ultra-processed food products (UPFP) by consumers through the information available on their labels. A cross-sectional study was carried out using data from the list of ingredients and the nutrition facts panel on labels of processed foods and UPFP available in Brazilian supermarkets between April and July 2017, totalling 8436 food items assessed, of which 84·0 % were UPFP. Of the total, 62·7 % of the UPFP would have the FoPNL and 65·1 %, 37·9 % and 12·9 % had flavouring, colouring and non-sugar sweeteners, respectively. Combining criteria for the FoPNL with any one of the three cosmetic additives analysed, 45·9 % of the UPFP were identified, and when considering the presence of the FoPNL, flavouring, colouring or non-sugar sweeteners, the identification increased to 89·9 %. Results showed that the current FoPNL in Brazil does not facilitate the identification of UPFP. In this sense, labels that indicate the presence of food additives with cosmetic functions (which are UPFP markers) could be a public health strategy to reduce the consumption of UPFP. Currently, food labelling regulations in Brazil are not aligned with Brazilian Dietary Guidelines recommendations.

In 2020, Brazil took an important step to ensure consumers make more informed food choices^(1)^, aligned with the right to adequate information, with the approval of the nutrition food labelling regulation (Resolution of the Collegiate Board (*Resolução da Diretoria Colegiada* – RDC) no. 429/2020^(2)^ and Normative Instruction (*Instrução Normativa* – IN) no. 75/2020^(3)^). This regulation comprises food products packaged in the absence of consumers, that is, those that are not prepared for immediate consumption, such as processed foods and ultra-processed food products (UPFP), although the regulation does not mention these groups. It includes, for added sugar, saturated fat and sodium, a ‘high in’ front-of-package nutrition labelling (FoPNL), in the format of a black and white rectangle with a magnifying glass, in products that exceed the cutoff from the nutrient profile model (NPM) of the National Health Surveillance Agency (*Agência Nacional de Vigilância Sanitária* – Anvisa).

Processed foods and UPFP are defined by the Nova food classification system^(4)^, which categorises food groups according to the extent and purpose of industrial processing. Nova classification is used in the Brazilian Dietary Guidelines of the Ministry of Health, with the recommendation of avoiding the consumption of UPFP^(5)^. To recognise this specific food product category, two types of ingredients can be considered markers to identify them: (1) food substances of no or rare culinary use and (2) classes of food additives whose function is to make the final product palatable or often hyper-palatable (food additives with cosmetic function, henceforth called as cosmetic additives), such as flavourings, flavour enhancers, colourings, emulsifiers, emulsifying salts, non-sugar sweeteners (NSS), thickeners, antifoaming, bulking, carbonating, foaming, gelling and glazing agents^(6)^.

Aligned with the discussion about industrial food processing, the Pan-American Health Organization (PAHO) proposes the integration of the Nova classification in the regulatory agenda, stating that food and beverage products evaluated with the PAHO NPM are limited to processed foods and UPFP, including the application for FoPNL regulations^(7)^. In Mexico, a warning related to the presence of NSS was included as mandatory information in the products^(8)^. Argentina, Mexico and Colombia approved FoPNL regulations aligned with the PAHO NPM, adopting its cutoffs. Other countries do not consider the Nova to identify products that must receive the warning and focus only on the critical nutrients in their FoPNL, independent of the Nova classification groups^(1,9)^. A more comprehensive FoPNL could be useful for better orienting consumers’ food choices.

Considering the scientific evidence from the last decade, which associates UPFP with negative health outcomes^(10–15)^, and the importance for consumers to easily recognise this food product category, this study aimed to explore combinations of the Brazilian FoPNL and/or three specific cosmetic additives (colourings, flavourings and NSS) in packaged foods and beverages marketed in Brazilian supermarkets, to strengthen the identification of UPFP by consumers through the information available on their labels.

## Methods

### Study type and sample

A cross-sectional study was carried out using data from the list of ingredients and nutrition facts panel on labels of packaged processed foods and UPFP available in Brazilian supermarkets. A total of ten outlets of major supermarket chains, located in areas with different family income levels, in two large Brazilian cities (São Paulo and Salvador) were selected for the study. Formal permission was sought from the supermarket chains before data collection was conducted. Details about the sample definition are available in Duran *et al.* ^(16)^.

### Data collection

Data collection took place between April and July 2017 and was conducted by trained researchers. Photographs were taken of each side of the package of all packaged foods and beverages available in the supermarket (around 14 000 products)^(17)^.

Data were entered manually by trained researchers on the RedCap online platform using a specific form developed together with the University of North Carolina at Chapel Hill in the USA and the Nutrition and Food Technology Institute (*Instituto de Nutrición y Tecnología de los Alimentos* – INTA) in Chile and adapted for use in the Brazilian study. As a reliability procedure, a subsample of 10 % of the records was typed twice (double data entry) by the same individual and by a second one to verify intra- and inter-observer reliability, respectively. Further details on the data collection process adopted were previously described by Duran *et al.* ^(16)^.

### Nova classification

All food items were independently classified into the groups (unprocessed or minimally processed foods, processed culinary ingredients, processed foods, UPFP)^(6)^ and subgroups of the Nova classification by a trained researcher (GCA), with experience in studies related to the classification. In this analysis, only processed foods and UPFP and their subgroups were considered.

### Front-of-package nutrition labelling identification and classification

After the exclusion of duplicate items, products without nutrition information, products available in more than one package size and products with multiple items, 11070 food products were considered in the data sample. Of these, only foods eligible to receive FoPNL were selected for this study. According to RDC no. 429/2020 published by Anvisa^(2)^, all foods and beverages packaged in the absence of consumers are eligible to receive the FoPNL, with the exception of fruits, vegetables, legumes, tubers, cereals, nuts, nuts, seeds, mushrooms, flour, meat and fish, eggs, cheese and fermented milk as long as they do not contain ingredients that add added sugars or significant nutritional value of saturated fats or sodium to the product. Also excluded from the FoPNL are milk, olive oil and other vegetable oils, salt, infant formulas, formulas for enteral nutrition, foods for weight control, dietary supplements, alcoholic beverages, products intended exclusively for industrial processing, products intended for food services, food additives and additives to food services. Among the sample, 8376 foods were considered eligible to receive the FoPNL. The nutrition information on labels (also called nutrition facts panel or nutrition declarations) was used to apply the criteria for high content of critical nutrients established by RDC no. 429/2020^(2)^ (Table 1). The classification was conducted by a trained researcher (GCA).

**Table 1.** The nutrient profile model (NPM) of the Brazilian front-of-package nutrition labelling (FoPNL) (RDC no. 429/2020)^2^

Nutrients	100 g solid foods	100 ml of beverages
Added sugar	≥ 15 g	≥ 7·5 g
Saturated fat	≥ 6 g	≥ 3 g
Sodium	≥ 600 mg	≥ 300 mg

### Food additives identification and classification

In this study, we focus on the assessment of flavourings, colourings and NSS. This decision was made based on a previous study indicating that flavourings and colourings were the most common cosmetic additives in Brazil, present in 47·1 % and 27·8 % of products, respectively^(18)^, while NSS were present in 10·8 % of products^(18)^. The inclusion of NSS is relevant in a scenario of potential reformulation due to the implementation of the ‘high in added sugar’ FoPNL.

All foods and beverages included in the study had their list of ingredients analysed item by item, in order to identify food additives, in accordance with the definition and classification of Anvisa^(19)^, as well as following the international numerical identification system of food additives in the list of ingredients, the International Numbering System, established by the FAO/WHO *Codex Alimentarius* Committee as an alternative to declaring the specific name of the additive^(20)^. Once the food additives were identified, they were classified according to their function, considering those with the functions of flavouring, colouring and sweeteners. The identification and classification of food additives were conducted by a trained researcher (VSPM). In these analyses, the occurrence of at least one of those cosmetic additives in the evaluated items was considered.

### Data analysis

In this study, only data from processed foods and UPFP, identified according to the Nova food classification system^(6)^, were explored.

The relative frequencies (%) of processed foods and UPFP and their subgroups with at least one critical nutrient in excess based on the Brazilian FoPNL and flavouring, colouring and NSS were estimated. Additionally, the relative frequency of ten different combinations of the presence of critical nutrients in excess and/or the three cosmetic additives (FoPNL or flavouring, FoPNL or colouring, FoPNL or NSS, FoPNL or flavouring or colouring, FoPNL or flavouring or colouring or NSS, FoPNL and flavouring and colouring and NSS, FoPNL and flavouring or colouring or NSS, FoPNL and flavouring, FoPNL and colouring, FoPNL and NSS) was also described.

## Results

Among the 8436 food items assessed, 16·0 % were classified as processed foods and 84·0 % as UPFP. Of the total, 55·4 % of the processed foods and 62·7 % of the UPFP would have FoPNL with some critical nutrient in excess, 65·1 %, 37·9 % and 12·9 % of the UPFP had flavouring, colouring and NSS, respectively, while 1·3 % of the processed foods had flavouring in their composition (Table 2).

**Table 2.** Proportion (%) of processed foods and ultra-processed food products available in Brazilian supermarkets with cosmetic additives (flavourings, colourings, non-sugar sweeteners (NSS)) and/or critical nutrients in excess based on the Brazilian magnifying glass (FoPNL), according to the Nova classification system

Food groups/food subgroups	Total sample (*n*)	FoPNL^‡^	Flavouring	Colouring	NSS	FoPNL or flavouring	FoPNL or colouring	FoPNL or NSS	FoPNL or flavouring or colouring	FoPNL or flavouring or colouring or NSS	FoPNL and flavouring and colouring and NSS	FoPNL and flavouring or colouring or NSS	FoPNL and flavouring	FoPNL and colouring	FoPNL and NSS
**Processed foods**	**1352**	**55·4**	**1·3**	**0·0**	**0·0**	**55·6**	**54·8**	**54·7**	**55·7**	**55·7**	**0·0**	**0·4**	**0·4**	**0·0**	**0·0**
Bread	60	23·3	1·7	0·0	0·0	25·0	23·3	23·3	25·0	25·0	0·0	0·0	0·0	0·0	0·0
Cheese	226	93·8	0·0	0·0	0·0	93·8	93·8	93·8	93·8	93·8	0·0	0·0	0·0	0·0	0·0
Salted/dried/ smoked meat	88	93·2	0·0	0·0	0·0	93·2	93·2	93·2	93·2	93·2	0·0	0·0	0·0	0·0	0·0
Others^*^	978	44·9	1·7	0·1	0·0	45·3	44·3	44·2	45·4	45·4	0·0	0·6	0·6	0·0	0·0
**Ultra-processed food products**	**7084**	**62·7**	**65·1**	**37·9**	**12·9**	**86·9**	**77·1**	**73·2**	**88·3**	**89·9**	**0·5**	**45·9**	**40·6**	**23·1**	**2·0**
Cold cuts and sausages	611	78·2	64·5	47·3	0·0	93·3	83·8	77·4	94·4	94·4	0·0	59·2	48·6	40·9	0·0
Sweet cookies	602	96·0	88·2	28·7	5·3	98·7	96·8	97·0	98·8	98·8	0·8	86·0	84·9	27·2	3·7
Salted biscuits	459	81·1	63·0	28·3	0·0	92·2	85·4	80·2	93·5	93·5	0·0	52·9	50·9	23·1	0·0
Margarine	53	96·2	67·9	88·7	0·0	98·1	98·1	96·2	98·1	98·1	0·0	90·6	66·0	86·8	0·0
Cakes and sweet pies	247	61·6	88·7	25·5	8·5	95·5	67·2	65·6	96·0	96·0	1·2	57·1	54·3	19·4	4·0
Bread	283	30·4	21·9	3·5	2·1	39·9	30·7	31·8	40·3	41·7	0·0	13·4	12·4	3·2	0·7
Sweets in general	1232	74·0	66·5	37·1	19·5	92·6	85·5	88·4	93·3	96·7	1·1	50·5	47·4	25·2	4·6
Carbonated beverages	105	53·4	93·3	67·6	44·8	96·2	81·9	95·2	98·1	99·0	1·9	49·5	49·5	38·1	1·9
Chocolate	233	100·0	91·0	7·3	10·3	100·0	99·6	99·6	100·0	100·0	0·4	90·6	90·6	7·3	10·3
Pizza, lasagne or pastry	356	48·6	44·1	43·5	0·0	65·4	63·2	48·3	71·1	71·1	0·0	34·0	27·0	28·7	0·0
Ready meals	387	35·9	50·0	37·7	0·0	68·0	60·7	35·7	72·1	72·1	0·0	23·3	17·6	12·7	0·0
Other sugary drinks	767	19·8	83·8	50·0	47·3	89·0	61·3	66·2	90·7	93·2	0·5	15·1	14·5	8·1	0·8
Dairy beverages	451	1·1	82·0	60·5	23·7	82·3	61·6	24·8	83·8	86·5	0·0	0·1	0·8	0·0	0·0
Ice cream	240	91·3	83·3	46·3	7·1	98·3	95·0	97·5	98·3	99·6	0·8	77·5	76·3	42·5	0·8
Sauces and spreads	632	73·4	54·0	32·6	1·4	84·2	77·7	73·3	86·6	86·6	0·6	51·4	42·9	28·0	1·3
Others^†^	381	95·0	13·4	40·9	0·0	95·3	96·1	94·8	96·6	96·6	0·0	43·6	12·9	39·6	0·0

* Salted/dried fish, dried seafood, canned fish, eggs, vegetables, pulses or cereals, olives, salted peanuts, fruit-based sweets and seasonings.

† Condiments and ready-made seasonings, ultra-processed cheeses and breakfast cereals.

‡ FoPNL classification considered: Added sugar: ≥ 15 g/100 g of solid foods or ≥ 7.5 g/100 ml of liquid foods; saturated fat: ≥ 6 g/100 g of solid foods or ≥ 3 g/100 ml of liquid foods; sodium: ≥ 600 mg/100 g of solid foods or ≥ 300 mg/100 ml of liquid foods.

When combining criteria for FoPNL and the presence of any one of the cosmetic additives analysed, 45·9 % of UPFP were identified. When considering criteria for FoPNL or the presence of one of the cosmetic additives, 89·9 % of UPFP were identified (Table 2).

Some specificities were found when considering the food groups: a high frequency of the FoPNL (100·0 % and 96·0 %) and flavouring (91·0 % and 88·2 %) in chocolates and sweet cookies, respectively; a high frequency of flavouring (83·8 %) and NSS (47·3 %) in other sugary drinks, which are products that already contain sugar in their composition; a high frequency of flavouring (82·0 %) and the presence of NSS (23·7 %) in dairy beverages, which are products that can contain sugar in their composition; and the presence of flavourings in some processed bread (1·7 %), which should not have this additive (Table 2).

Flavourings were very present in cakes and sweet pies (88·7 %) and ice cream (83·3 %) and colourings in margarine (88·7 %). NSS were mostly found in beverages (carbonated beverages (44·8 %), other sugary drinks (47·3 %) and dairy beverages (23·7 %)). Carbonated beverages were frequently composed of all three cosmetic additives (flavouring (93·3 %), colouring (67·6 %) and NSS (44·8 %)) (Table 2).

When considering the presence of FoPNL or any of the cosmetic additives analysed, all the UPFP subgroups presented percentages higher than 71·0 %, with the exception of breads. The main UPFP subgroups were chocolate (100·0 %), ice cream (99·6 %), carbonated beverages (99·0 %), sweet cookies (98·8 %) and margarine (98·1 %). The presence of FoPNL and flavouring, colouring or NSS was higher in chocolate and margarine (90·6 % each), sweet cookies (86·0 %) and ice cream (77·5 %) (Table 2).

## Discussion

In the present study, more than 8000 processed foods and UPFP available in Brazilian supermarkets were described in relation to the presence and combinations of FoPNL and/or specific food additives with cosmetic functions. As a group, UPFP would receive FoPNL more frequently than processed foods (62·7 % *v*. 55·4 %, respectively), but there are important differences considering the subgroups of both groups. More than 90 % of processed cheese and salted/dried/smoked meat would receive FoPNL, while about only 30 % of ultra-processed breads, 20 % of ultra-processed sugary drinks and 1 % of ultra-processed dairy beverages would.

Considering the presence of critical nutrients, it is notable the difference in the frequency of products that would receive the FoPNL comparing the Brazilian criteria, presented in this study, and the PAHO criteria, which is considered the golden standard for the region since it is applied specifically for processed foods and UPFP and has more restrict cutoffs. Using the same data from this study and the PAHO criteria, 90·9 % of processed foods and 97·1 % of UPFP items had at least one critical nutrient in excess^(21)^. Based on the comparison of our results with the previous one^(21)^, it is clear that if Brazil’s policy adopted the PAHO criteria, it would capture more UPFP than using Anvisa’s nutrient profile.

Using a different analytical approach and the NPM adopted in the Chilean FoPNL policy, it was identified that in the USA, 33 % of food items purchased by North American households were UPFP and high in saturated fat, sodium or sugar (HFSS), and 16 % were UPFP but were not higher in critical nutrients (according to the Chilean NPM, which is not in line with the gold standard NPM of PAHO)^(22)^. In the UK, using the NPM from their Food Standards Agency, the classification of HFSS captured half of UPFP based on all foods consumed and percent of energy, but only about a third based on food weight. In this context, the most common UPFP that was not HFSS was low-calorie soft drinks and white bread^(23)^.

In this study, combining the information about critical nutrients and three common types of cosmetic additives (flavouring or colouring or NSS), almost 90 % of the UPFP contained at least one of them. Using PAHO criteria and the presence of cosmetic additives, this value reached 98·8 %^(21)^. In the USA, the combination of HFSS and colouring, flavouring and NSS was present in 66 % of the items purchased by families and HFSS plus twelve classes of additives in 73 %^(22)^. These results can indicate that the use of some cosmetic additives is useful but not sufficient to identify some UPFP when an NPM is not aligned with PAHO’s gold standard. In this case, the identification of UPFP would depend on the presence of food substances of no or rare culinary use, which is more difficult to be assimilated by the population^(6)^. All these results indicate that clear information about critical nutrients and cosmetic additives on the labels can contribute to the identification of UPFP by consumers and consequently to better food choices. Considering our results and the literature^(21,22)^, the first step for Brazil would be to adopt the PAHO’s NPM, which focuses on the application to processed foods and UPFP, has more restrict cutoffs than Brazilian legislation and includes NSS, since it would allow the policy to capture more UPFP. In the sequence, it is also relevant to incorporate cosmetic additives to close the regulatory gap even further and allow people to differentiate UPFP from processed food with an excess of critical nutrients. A future goal to improve the food labelling system can be the inclusion of a specific warning for UPFP, but it seems to be far from the current regulatory discussion.

With regard to NSS, in our study and in the North American study^(22)^, the inclusion of this food additive in the analyses did not result in a higher percentage of items that would be targeted for policy intervention. Trends in other countries, such as Chile, have indicated that reformulation due to FoPNL may lead to increases in NSS in the food supply^(24,25)^. In this sense, it is an important element of monitoring in future studies about the implementation of FoPNL in Brazil.

In Brazil, food labelling standards are insufficient to promote a clear identification of UPFP by consumers, even though the Brazilian Consumer Defense Code (*Código de Defesa do Consumidor*)^(26)^ requires that the products should adequately declare the specification of quantity, characteristics, composition and quality of the different products available in the country. There are three main mandatory standards that regulate the ultra-processing markers evaluated in this study: the RDC no. 429/2020^(2)^ and the IN no. 75/2020^(3)^, which determine the FoPNL declaration, and the RDC no. 727/2022^(27)^, which regulates the list of ingredients. The FoPNL is an improvement for access to information on labels; however, it only highlights the high level of specific critical nutrients, while there are no label systems that indicate the presence of food additives with cosmetic function or the industrial processing level of the product.

Furthermore, the Brazilian FoPNL system does not identify the wide range of UPFP available on the food supply, particularly sweetened beverages. For instance, only 1 % of the dairy beverages carry a FoPNL about their high content of critical nutrients, despite over 80 % of these products containing flavourings. Similarly, less than 20 % of other sugary drinks are flagged by the current FoPNL system, while more than 80 % of them contain at least one flavouring. Highlighting the presence of colourings, flavourings and NSS on food packaging is even more important for those subgroups in which the current labels do not identify as unhealthy. This labelling information can empower consumers to make better-informed decisions about the foods they consume^(28)^.

The list of ingredients is organised in descending order, and the food additives in general are positioned at the end, regardless of their quantity. In contrast to the FoPNL, the list of ingredients does not have design specifications (format, colour, font size, name, etc.) for its declaration. The lack of standardisation contributes to the difficulty of identifying the presence of cosmetic additives, such as colourings, flavourings and NSS^(29)^ in packaged foods and beverages. The identification of flavourings has a specificity because it is not mandatory to declare the type of flavour used in the product and the industry can just inform terms such as ‘flavour of’ and ‘flavouring’^(18)^. Flavourings were also identified in bread classified as processed foods, which should not have any cosmetic additive in their composition.

An important aspect is that food additives can serve multiple functions, which can complicate the classification of foods within processed and ultra-processed. Since the classification is made considering the main function of food additives, it serves as a useful reference, but it has limitations in identifying processed foods and UPFP. For instance, citric acid may function as an antioxidant (a non-cosmetic use) or as a colour retention agent (a cosmetic use). Brazilian legislation does not require manufacturers to specify the function of an additive on product labels. Additionally, the Codex Alimentarius classifies some minimally processed ingredients, such as curcumin, turmeric, paprika and hibiscus, as food additives. In this study, we adopted a conservative approach, identifying all additives with flavouring, colouring and sweetening functions, even if they were not necessarily used for those specific purposes in the product. As a result, some processed foods in the study contained cosmetic additives, but they are exceptions.

It is important to note that the presence of cosmetic additives is one of the markers of UPFP, but not the only determining factor. The presence of flavourings or colourants must be assessed in the context of each product. If an additive is used to significantly modify a product’s taste, texture or appearance – transforming it into an artificially formulated food – it may be classified as ultra-processed. However, if the additive is natural or does not significantly alter the food matrix, the product may still be classified as processed, since the Nova classification categorises foods based on the extent and purpose of industrial processing^(6)^.

This situation strengthens the need to monitor food label information to capture changes adopted by the food industry over time, such as items that traditionally were processed becoming ultra-processed due to changes in their composition. Besides that, an obstacle that makes it difficult to identify all cosmetic additives for consumers is the possibility of declaration through the International Numbering System, rather than its function or specific term^(27)^.

It is important to highlight that the analysed cosmetic additives are approved by Anvisa and, in theory, are considered safe, since the acceptable daily intake is respected. However, two aspects need to be considered. First, the safety of food additives is based on toxicological studies, like genotoxic, carcinogenic and teratogenic effects, but little is considered about other possible impacts on health, which have been described in the literature, such as behavioural disorders and common mental disorders, hypersensitivity and autoimmunity reaction and metabolic and inflammatory changes^(30–39)^. Furthermore, safety analyses of these food additives are carried out with them being considered in isolation; that is, each one is evaluated separately. However, this is not the current context in which these compounds are used, as there are ‘mixtures’ of food additives, mostly cosmetic, in a single UPFP, and even more mixtures in the context of ultra-processed meals. In a previous study, using the same data but including all food products available in supermarkets, it was identified that almost 70 % of food items had two or more food additives^(18)^. These considerations, inserted in the current moment of food transition, in which there is an increase in the consumption of UPFP, highlight the need to reevaluate the safety and use of these food additives in the current scenario.

To promote a healthy food environment aligned with the recommendations of the Brazilian Dietary Guidelines, it is essential that public policies address various aspects of its principles and recommendations, supporting the population in making healthy food choices. The Brazilian Dietary Guidelines advise against the consumption of UPFP because, in addition to their poor nutritional composition – often high in sugar, saturated fat and sodium – these products contain cosmetic additives like colourings, flavourings and NSS that can negatively impact health^(6)^. Considering the potential harm of these cosmetic additives, it is crucial to highlight their presence in the composition of foods and beverages, providing consumers with the information needed to make healthy food choices. There are two improvements on the current Brazilian food labelling regulation about food additives that could be done: highlight in bold the colourings, flavourings and NSS in the list of ingredients; and include a front-of-package warning label for the presence of these ingredients. Additionally, the inclusion of an ‘ultra-processed food product’ front-of-package warning label could be an important advance^(40)^.

This study has some limitations. It did not cover all processed foods and UPFP available in the Brazilian retail market, considering the focus was exclusively on some supermarket chains. It implies that food products sold only by other food outlets and products of their own brand by other supermarket chains were not included. On the other hand, as strengths of the study, we can mention the inclusion of supermarkets from different chains, placed in different Brazilian regions and income levels areas, and also supermarkets that are responsible for more than half of total food energy and UPFP energy purchased in Brazil^(41)^, and all packaged foods and beverages available were included in the study, resulting in a large sample of products analysed. We also consider a strength of the study that information on only three cosmetic additives could improve the possibility of identifying UPFP. It is useful for food labelling but also for health promotion strategies.

We conclude that a combination of the FoPNL with flavouring, colouring or NSS, important cosmetic additives, can contribute to better identification of UPFP by consumers since the Brazilian food labelling regulation is insufficient to help consumers in the rapid identification of UPFP. Improving the NPM used in the regulation as well as the visibility of cosmetic additives on labels could be a public health strategy to allow healthier food choices following the Brazilian Dietary Guidelines, which recommends avoiding the consumption of UPFP.
